# 

**Published:** 1856-09

**Authors:** 


### RECEIPTS ON SUBSCRIPTIONS,

FROM JUNE 23D TO AUG. 17TH, 1856.

**ILLINOIS.**

| | | |
|---|---|---|
| Dr. R. J. Stough, - | Vol. 4, | $2,00 |
| " J. S. Miller, - | " 5, | 2,00 |
| " E. E. Rogers, - | " 5, | 3,00 |
| " A. Parronett, - | " 5, | 2,00 |
| " E. R. Willard, - | " 5, | 2,00 |
| " S. O. Long, - | " 6, | 2,00 |
| " L. A. Engle, - | " 5, | 2,00 |
| " A. Mahan, - | " 5, | 2,00 |
| " J. W. Waters, - | " 5, | 2,00 |
| " A. C. Buffum, - | " 5, | 2,00 |
| " A. W. Butler, - | " 5, | 2,00 |
| " Geo. Ryan, - | " 5, | 2,00 |
| " John Niglas, - | " 5, | 2,00 |
| " R. S. Molony, - | " 5, | 2,00 |
| " G. H. Scott, - | " 4, | 2,00 |
| " J. P. H. Stevenson, | " 5, | 2,00 |
| " A. Hull, - | " 5, | 2,00 |
| " N. G. Stevens, - | " 5, | 2,00 |
| " H. Shoemaker, - | " 5, | 2,00 |

**INDIANA.**

| | | |
|---|---|---|
| Dr. J. Miller, - | Vol. 5, | $2,00 |
| " S. Ross, - | " 4, | 2,00 |
| " J. C. Helm, - | " 5, | 2,00 |
| " J. P. Terrell, - | " 5, | 0,50 |

**IOWA.**

| | | |
|---|---|---|
| Dr. H. C. Rawson, - | Vol. 5, | $1,00 |
| " C. W. Davis, - | " 3&4, | 4,00 |
| " J. S. M. C. Van Ness, | " 5, | 2,00 |

**WISCONSIN.**

| | | |
|---|---|---|
| Dr. Wm. Carger, - | Vol. 5, | $2,00 |
| " John Cowen, - | " 5, | 1,00 |
| " J. Philips, - | " 4&5, | 4,00 |
| " S. C. Johnson, - | " 5, | 2,00 |
| " H. A. Yeomans, - | " 5, | 2,00 |

**MICHIGAN.**

| | | |
|---|---|---|
| Dr. W. M. Avery, - | Vol. 5. | $2,00 |

**MINNESOTA TERRITORY.**

| | | |
|---|---|---|
| Dr. LaFayette Redman, | Vol. 5, | $2,00 |

